# Intensity modulated radiotherapy (IMRT) in the treatment of children and Adolescents - a single institution's experience and a review of the literature

**DOI:** 10.1186/1748-717X-4-37

**Published:** 2009-09-23

**Authors:** Florian Sterzing, Eva M Stoiber, Simeon Nill, Harald Bauer, Peter Huber, Jürgen Debus, Marc W Münter

**Affiliations:** 1 Department of Radiation Oncology, University of Heidelberg, Heidelberg, Germany; 2 Clinical Cooperation Unit Radiation Oncology, German Cancer Research Center (dkfz), Heidelberg, Germany; 3 Department of Anaesthesiology, University of Heidelberg, Heidelberg, Germany

## Abstract

**Background:**

While IMRT is widely used in treating complex oncological cases in adults, it is not commonly used in pediatric radiation oncology for a variety of reasons. This report evaluates our 9 year experience using stereotactic-guided, inverse planned intensity-modulated radiotherapy (IMRT) in children and adolescents in the context of the current literature.

**Methods:**

Between 1999 and 2008 thirty-one children and adolescents with a mean age of 14.2 years (1.5 - 20.5) were treated with IMRT in our department. This heterogeneous group of patients consisted of 20 different tumor entities, with Ewing's sarcoma being the largest (5 patients), followed by juvenile nasopharyngeal fibroma, esthesioneuroblastoma and rhabdomyosarcoma (3 patients each). In addition a review of the available literature reporting on technology, quality, toxicity, outcome and concerns of IMRT was performed.

**Results:**

With IMRT individualized dose distributions and excellent sparing of organs at risk were obtained in the most challenging cases. This was achieved at the cost of an increased volume of normal tissue receiving low radiation doses. Local control was achieved in 21 patients. 5 patients died due to progressive distant metastases. No severe acute or chronic toxicity was observed.

**Conclusion:**

IMRT in the treatment of children and adolescents is feasible and was applied safely within the last 9 years at our institution. Several reports in literature show the excellent possibilities of IMRT in selective sparing of organs at risk and achieving local control. In selected cases the quality of IMRT plans increases the therapeutic ratio and outweighs the risk of potentially increased rates of secondary malignancies by the augmented low dose exposure.

## Background

In more than a decade of clinical Intensity Modulated Radiation Therapy (IMRT) this method of high precision radiotherapy has proven remarkable advances in target conformity, dose escalation in the target volume and sparing of neighbouring organs at risk [1-14]. These qualities permit the irradiation of patients with complex shaped tumors at problematic locations which could not be treated successfully with conventional radiation methods. Within IMRT again different technical solutions are being used. They all have the principle in common that radiation beams with different intensities are used depending on how much tumor or organ at risk is located within different areas of the beam. This way dose distributions can be adapted to irregular tumor geometries close to organs at risk. It is a rather difficult task to produce irregular intensity maps with a linear accelerator that is designed to produce beams of homogeneous intensity. A very common approach is segmental MLC-IMRT (step-and-shoot-IMRT) [1]. The irregular fields are created as a summation of many small fields resulting in a pulsed dose application. Another way to modulate intensity is the dynamic movement of collimator leaves during beam application which is called dynamic MLC-IMRT or sliding window technique [15]. A third common technique is helical tomotherapy that uses a rotational beam delivery in a helical fashion together with a binary collimator [16]. With all these devices excellent treatment options can be opened for the most challenging cases in radiation oncology. Examples are parotid gland sparing in head-and-neck tumors or spinal cord sparing for tumors of the vertebral column.

The history of IMRT for children is markedly different to the history of IMRT for adult patients. While IMRT for adults is a widely used as a standard of care for many indications meanwhile, for several reasons IMRT was used with great caution in the paediatric population. Among these are increased fraction time, necessity for exact immobilization with tailor-made steep dose gradients present and the fear of increased secondary malignancy induction by changes in low dose spillage or integral dose [17-21].

This study describes experience and outcome of IMRT for children and adolescents in our institution. In addition a review of the available literature reporting on technology, quality, toxicity, outcome and concerns of IMRT is given.

## Methods

When radiotherapy is required for children within a multimodal study protocol, in our institution first planning with conventional techniques is performed. If problems with target coverage or sparing of close organs at risk occur, IMRT is evaluated for potential benefits in this regard.

From 1999 through 2008, at the German Cancer Research Center, 31 children and adolescents with a mean age of 14.2 years (range 1.5 - 20.5 years) were treated using IMRT. 17 patients were female, 14 were male. 21 patients were less than 18 years old. In total, the treated group consisted of twenty different tumor histologies, with Ewing's sarcoma being the largest group (n = 5), followed by juvenile nasopharyngeal angiofibroma, esthesioneuroblastoma and rhabdomyosarcoma with three patients each. Table 1 shows more detailed information about the patients' characteristics. Treatment location was head and neck in 50% of the treated sites (n = 17), other treatment locations were abdominopelvic (n = 5), intracranial (n = 3), thoracic wall (n = 5) and spine (n = 4). 28 patients were treated with curative intent despite most patients having advanced or even metastatic (cases #2, #4, #23, #30) disease. Eighteen patients underwent IMRT as part of multimodality therapy, e.g. as part of a protocol. Eleven patients received adjuvant radiotherapy and two patients radiotherapy only (cases #29, #7). One boy with alveolar rhabdomyosarcoma of the nasal cavity was treated twice due to local relapse (case #23). One adolescent with a desmoplastic small cell tumor was treated three times at different sites (case #12).

**Table 1:** Patient characteristics

**Case**	**Diagnosis**	**Location**	**Age** **[years, months]**	**# fields**	**median Dose** **[Gy]**	**number of fractions**	**Previous RT**
1	Ewing's sarcoma	orbita	14, 7	9	54	30	

2	Ewing's sarcoma	spine (cervical)	15, 0	7	45	25	RT pelvis 45 Gy

3	Ewing's sarcoma	infratemporal fossa	15, 4	7	54	30	

4	Ewing's sarcoma	pelvis	16, 10	8	54	30	

5	Ewing's sarcoma	scapula	19, 9	9	45	25	

6	Myoepithelial Parotis Ca	parotid gland	19, 1	7	66	33	

7	Giant cell tumor	os sacrum	20, 6	7	66	33	

8	Meningeoma	intracranial	12, 4	7	57.6	32	

9	Desmoid Tumor	spine (cervical)	17, 7	7	54	30	

10	Aggressive fibromatosis	thoracic wall	19, 8	5	45	25	RT thoracic wall 28.8 Gy

11	Angiofibromatous tumor	spine (cervical)	19, 1	7	56	28	

12	Desmoplastic small cell tumor	abdomen	17, 3	7	56	28	
		
		abdomen	18, 1	7	45	25	
		
		thoracic wall	19, 3	7	50.4	28	

13	Adenoid cystic carcinoma	parotid gland	17, 0	7	66	33	

14	Astrocytoma WHO III	intracranial	16, 0	8	30.6	17	RT neurocranium 5.4 Gy + TBI 12Gy, RT right hemisphere 54 Gy

15	Malignant opticus glioma	optic nerve	4, 5	7	50	25	previous iodine seed implantation

16	Lymphoepithelial Carcinoma	nasopharynx	17, 11	9	66	30	

17	Melanoma	orbita	7, 6	8	60	30	

18	Juvenile nasopharyngeal fibroma	nasopharynx	10, 11	7	50.4	28	

19	Juvenile nasopharyngeal fibroma	nasopharynx	15, 11	7	50.4	28	

20	Juvenile nasopharyngeal fibroma	nasopharynx	18, 5	7	50.4	28	

21	Rhabdomyosarcoma	thoracic wall	5, 0	7	21.6	12	

22	Rhabdomyosarcoma	abdomen	18, 2	7	45	25	

23	Rhabdomyosarcoma	neck	4, 9	7	45	25	
		
		neck (re-Rt)	7, 4	7	36	20	

24	Esthesioneuroblastoma		15, 10	10	60	30	

25	Esthesioneuroblastoma		17, 10	7	54	30	

26	Esthesioneuroblastoma		18, 6	7	63	32	

27	PNET	thoracic wall	1, 6	7	41.4	23	

28	PNET	thoracic wall/spine	19, 5	7	54	30	

29	Chondrosarcoma	scull	16, 3	7	64	32	

30	Neuroblastoma	adrenal gland	3, 4	8	39.6	22	

31	Hypopharynx-ca	neck	4, 9	5	60	30	

Three patients had previously received standard external beam radiation (cases #2, #10, #14), including a girl with metastatic Ewing's sarcoma, after definitive treatment with multiagent chemotherapy and radiotherapy of the pelvis. This girl received IMRT for tumor recurrence involving the cervical spine. The second patient, a 19-year old male with aggressive fibromatosis of the thoracic wall started radiation treatment two years ago, but declined further treatment after an administered total dose of 28.8 Gy at that time. He received IMRT to the previously treated site. The third patient, a 16-year old boy underwent radiotherapy of the neurocranium (total dose 5.4 Gy) six years ago as part of multimodality treatment of an acute lymphoblastic leukaemia. About four years later he presented with an anaplastic astrocytoma and therefore received external beam radiation to the right hemisphere (total dose 54 Gy). IMRT was delivered sixteen months later for recurrent astrocytoma.

One girl with malignant optical nerve glioma was treated with an iodine seed implantation four years prior to IMRT (case #15).

Administered doses varied according to whether IMRT was definitive, postoperative, delivered to a previously treated tumor site, or part of a treatment protocol (e.g. Ewing's sarcoma) and depended on the proximity of critical organs.

Follow up examinations including MRI scans were performed six weeks after completing radiotherapy and after that in intervals of three to six months for the first two years. Further follow-up visits usually took place annually.

### Radiotherapy

Inverse treatment planning for stereotactic-guided IMRT was realized by the KonRad treatment planning system, developed at our institute [8,22]. The KonRad system is connected to the 3D treatment planning system VIRTUOS, which allows calculation and visualization of the dose distribution. 3D planning based on contrast enhanced MRI and CT imaging was performed, using individually manufactured rigid scotch masks for head immobilization. Thoracic and abdominopelvic targets were positioned with a vacuum bag and a scotch cast mask fixation. Definition of the planning target volume was performed on the basis of image fusion techniques. In most patients IMRT was administered using a simultaneous integrated boost concept.

A Siemens linear accelerator (Medical Solutions Siemens, Erlangen, Germany) with 6 MV photons was used for treatment. It is equipped with an integrated motorized multileaf collimator, which allows a sequential step-and-shoot technique. In three patients (cases #17, #26, #27) a miniature-multileaf collimator (ModuLeaf MLC, MRC-Systems GmbH, Heidelberg, Germany) with a leaf width of 2.75 mm at isocenter was used. This collimator is attached to an accessory holder of the Siemens accelerator.

During treatment all patients were evaluated at least on a weekly basis to assess acute toxicity.

## Results

Median follow up time was 34 (1 - 68) months; mean administered dose was 51.6 Gy (21.6 - 66), including the patients that received concomitant chemotherapy. The two patients previously treated with standard external beam radiation on the IMRT treatment site, were treated up to a total dose of 45 Gy and 30.6 Gy respectively (cases #10, #14).

Intravenous sedation with propofol during radiotherapy session was necessary in 6 children (cases #15, #21, #23, #27, #30, #31). These children were all younger than 6 years at the time of treatment This was tolerated well without severe side-effects and with fast recovery after treatment. No general anaesthesia with intubation was necessary.

### side effects

Reported acute side effects of radiotherapy were low grade skin erythema (CTC grade I-II), mucositis (CTC grade I-II), local alopecia, mild nausea, mild diarrhoea, loss of taste and epistaxis (case #19). Pancytopenia occurred in four patients (cases #1, #2, #4, #28) who received concomitant chemotherapy. In two of them pancytopenia (CTC grade III) resulted in treatment interruption for two days. No other severe acute side effects were observed.

**Figure 1:**
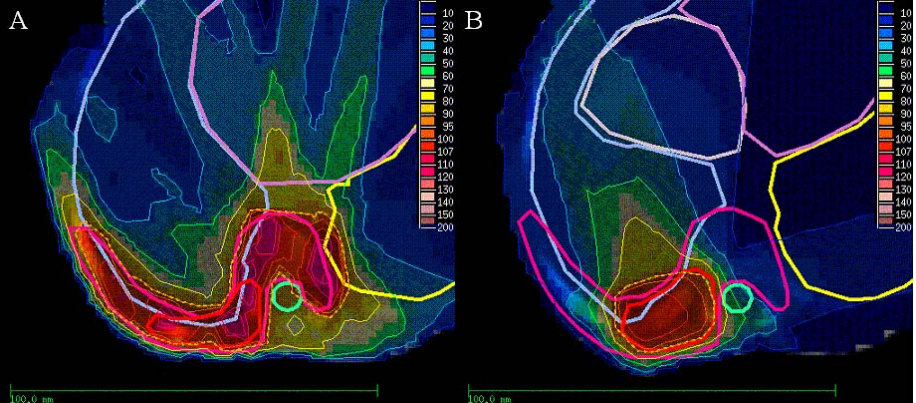
**IMRT-Plan for treatment of a 1.5 year old boy with a primitive neuroectodermal tumor (PNET) of the right thoracic wall**. **A: **A prescribed dose of 30.6 Gy to the PTV. **B: **41.4 Gy prescribed to the boost. IMRT-Plan in colour wash shows the 90% isodose region (dotted line).

One patient developed thoracic scoliosis two years following spine irradiation (case #27, figure 1). One adolescent, who was also treated with chemotherapy, claims of hypoaesthesia in his right forearm, two years after upper thoracic wall irradiation (case #28). One girl developed slight enophthalmia after irradiation for a Ewing's sarcoma of the orbit, visual acuity though is not impaired (case #1). No other late toxicity was observed so far among survivors.

Figure 1 displays the treatment plan for a 18 months old boy (case #27) with primitive neuroectodermal tumor (PNET) of the right thoracic wall. He received chemotherapy according to the Euro Ewing 99 protocol followed by tumor resection with positive pathological margins. Postoperative IMRT was delivered in order to decrease the dose to the nearby spinal cord and lungs with a median prescribed dose of 30.6 Gy to the PTV and 41.4 Gy to the boost. During the radiation course regular CT-scans with an in-room CT-Scanner were performed to confirm correct patient position. Thirty-eight months after finishing treatment he underwent surgery for straightening of thoracic scoliosis. This occurred inspite of inclusion of the complete vertebral body in the PTV. An asymmetric growth of the thoracic wall is a possible explanation for this.

**Figure 2:**
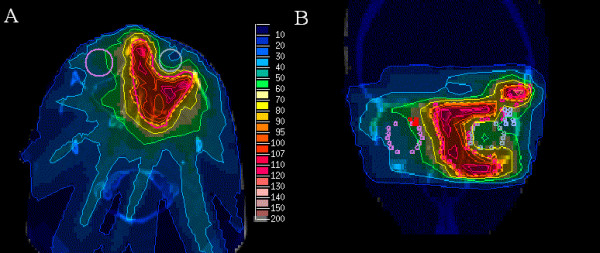
**IMRT-plan for treatment of a 14 year old girl with Ewing sarcoma of the left orbit with a median prescribed dose of 54 Gy**. **A: **Axial view of the dose distribution in colour wash shows the 90% isodose region (dotted line). **B: **Coronal view of the dose distribution with sparing of the eye.

Figure 2 shows the IMRT plan for a 14 year old girl (case # 1) with a Ewing's sarcoma of the left orbit, infiltrating the dura mater and the left ethmoid sinus. The patient received multiagent chemotherapy (7 cycles VIDE (vincristine, ifosfamide, doxorubicin, etoposide) followed by 6 cycles VAC (vincristine, adriamycin, cyclophosphamide)) and tumor resection (R1) prior to IMRT treatment. IMRT was delivered in order to spare the lacrimal gland, optic nerve and eyeball. At present, there are no signs of tumor recurrence with an actuarial follow up of four and a half years. Visual acuity is 1.0 on both eyes, though the patient developed slight enophthalmia on the treated site.

### local control and survival

Local failure occurred in 10 of 31 patients (table 2), time to local failure was 4 - 53 months. In the event of local tumor progression patients received chemotherapy or surgical tumor resection, one patient with carcinoma of the hypopharynx was reirradiated using IMRT (case #31). No local relapse occurred among patients with juvenile nasopharyngeal fibroma and esthesioneuroblastoma. So far, 5 patients died due to distant metastases (cases #30, #31, #21, #5, #4).

**Table 2:** Local failure after IMRT

**Case**	**Diagnosis**	**Time to local failure [months]**	**Dose** **[Gy]**	**Treatment following failure**
2	Ewing sarcoma	7	45	chemotherapy

4	Ewing sarcoma	9	54	chemotherapy

6	myoepithelial Parotis-carcinoma	7	66	surgery

8	Meningeoma	53	57.6	surgery

9	Desmoid tumor	14	54	surgery

11	Angiofibromatous tumor	7	56	surgery

15	Optic nerve glioma	36	50	surgery

21	Rhabdomyosarcoma	8	21.6	chemotherapy

23	Rhabdomyosarcoma	29	45	chemotherapy

31	Hypopharynx-Carcinoma	4	60	Re-irradiation (IMRT)

## Discussion

We present a very heterogeneous group of children and adolescents with 20 different tumor entities. All of these 31 patients have a very complex oncological constellation in common that made the application of a sufficient radiation dose extremely difficult with conventional radiotherapy techniques. Here the possible benefits of IMRT like the sparing of organs at risk and the possibility of dose escalation were considered to be more important for the treatment success than the potentially increased risk of secondary malignancies. We tried to increase chances of cure the patients accepting possible risks in a matter of decades in case of success. IMRT was feasible even if anaesthesia was necessary and resulted in good local control rates for this group of children who represents a selection of extraordinary and difficult cases.

IMRT could be applied with only few low grade acute toxicities and hardly any long term side effects so far. It is important to note that the follow up is still quite short to assess secondary malignancies. This radiotherapy technique allows reirradiations in difficult localisation that could not be performed safely before.

In contrast to the big amount of publications in treating adult patients with IMRT, there is only few data in literature about the use of IMRT in the paediatric population. Good experiences with the treatment of twenty-two children with IMRT have been reported by Bhatnagar et al. [23]. They described substantial sparing of surrounding critical structures in cranial, abdominopelvic or spinal lesions, altogether a selection of very difficult oncological situations. Conventional treatment technologies would have resulted in a markedly higher dose to organs at risk or would have required compromises regarding the possible target dose.

Penagaricano et al. summarized their experience of 5 children treated with IMRT with a high degree of conformality [24]. The dose distribution could be adapted to arc shaped volumes in contrast to conventional therapy where treated volumes are usually box shaped and encompass big areas of treated normal tissue. Similar conclusions are drawn by Paulino et al. in their synopsis of this method for children [24,25]. They summarize that IMRT is a valuable alternative to conventional treatment techniques for paediatric cancer patients. The improved dose distributions coupled with the ease of delivery of the IMRT fields make this technique very attractive, especially in view of the potential to increase local control and possibly improve on survival. A third survey of a heterogeneous group of children treated with IMRT is given by Teh et al. within a general article about decreased treatment related morbidity with IMRT [26]. Experiences with 185 patients treated with IMRT in general are presented, among these forty children suffering from different tumors. Similar to the conclusions by the authors described before they conclude that IMRT offers new options in escalating dose and achieving better local control while simultaneously reducing toxicity.

Besides these compilations of composed cohorts a larger number of articles provides data on special indications and more predefined collectives. They specially deal with intracranial or head-and-neck tumors since the sensitive structures like eyes, brain stem, parotid glands or inner ears represent an extraordinary challenge in the radiotherapeutic management. Starting with the biggest of all central nervous treatments the irradiation of the entire craniospinal axis as required in medulloblastoma or germinoma can be done with improved conformity and sparing of sensitive structures as shown by Penagaricano et al. [27]. In a retrospective planning evaluation they illustrate the possibilities of helical tomotherapy (as one solution of IMRT) to cover a target volume of this size avoiding the problems of field junctions and the resulting dangers of under or overdosage inherent in conventional techniques. After treating the whole craniospinal axis the primary tumor region is supposed to be irradiated with an extra boost to the posterior fossa. Huang et al. describe reduced ototoxicity when sparing the inner ear by IMRT compared to conventional radiotherapy, where the cochlear region receives the full therapeutic dose [28]. Thirteen percent of the IMRT Group had grade 3 or 4 hearing loss, compared to 64% of the conventional-RT group. The sparing of the hearing apparatus is of special importance since several modern combined chemotherapy regimens contain ototoxic agents like cisplatinum. Jain et al. showed that this improvement of ototoxicity was not achieved at the cost of increased neuropsychological changes [29].

Another challenging situation in that IMRT might substantially improve the treatment is retinoblastoma. Krasin et al. presented a planning study comparing different conventional photon, electron and IMRT techniques in the treatment of intraocular retinoblastoma [30]. The best sparing of the bony orbit was achieved with IMRT yielding a promising potential of avoiding asymmetrical bone growth after successful radiotherapy. The mean volume of bony orbit treated with IMRT above 20 Gy (as a threshold of bone growth disturbance) was 60% in contrast to 90% in conventional technique. Schroeder et al. report on 22 children with localized intracranial ependymoma treated with IMRT. They were able to achieve a three year local control of 68% while enabling minimal rates of toxicity (no visual or hearing impairment, no necrosis, no myelitis) [31].

The irradiation of head-and-neck tumors is quite rare in children. Nevertheless long term toxicity is a huge concern and often impairs the quality of life. Special focus here is xerostomia caused by a fibrotic atrophy of the parotid glands. Consecutive dental damage, dysphagia, problems of speach and taste are feared. In a study by Wolden et al. twenty-eight patients with head-and-neck rhabdomyosarcoma were treated with IMRT. The age ranged from 1-29 years, the thee year local control was 95% with minimal side effects [9]. In a similar approach by the groups of Atlanta (20 children) and Houston (19 children) head-and-neck rhabdomyosarcomas could be treated with a 3 year local control of 100% and a four year local control of 92.9% respectively [32,33]. Combs et al. presented a cohort of 19 children with rhabdomyosarcoma treated with stereotactic radiotherapy (n = 14) or IMRT (n = 5) [34]. The three and five-year local control rate was 89%, no toxicity > CTC grade 2 were observed. An Indian analysis of IMRT for nasopharyngeal cancer (19 children) showed reduced toxicity in terms of xerostomia, skin reaction and mucous membrane reaction compared to conventional radiotherapy (17 children) [35]. Acute xerostomia grade 2 occurred in 31.6% in IMRT vs. 88.2% in conventional radiotherapy. Grade 2 dysphagia was also significantly reduced with 42.0% vs. 94.1%. IMRT was also able to provide superior target coverage and as a consequence of the reduced toxicity an improved compliance.

Juvenile angiofibroma can be cured by radiotherapy in unresectable or relapsing cases. They are difficult to treat for because of the same surrounding risk structures as discussed above. Especially with respect to the benign nature of these tumors a well balanced toxicity profile is vital as described by Kuppersmith et al. and can be achieved by the means of IMRT [36].

Another potential indication is the radiosurgical treatment of arteriovenous malformations (avm). Lesions that are unresectable and not accessible for interventional neuroradiology can be obliterated by high dose single course radiotherapy. Fuss et al. presented the possibilities of IMRT in seven children with avm of complex shape, that could hardly be treated with conventional methods [37]. Two avm obliterated completely, three partially, while no treatment related side effects occurred.

In the discussions about precautions of IMRT in children the advantages are achieved at the cost of raised low dose outside the target. With a higher number of monitor units required the total body dose can increase significantly [38]. However, in a study by Koshy et al. no increased extra target dose to thyroid, breast, and testis was seen in children treated with IMRT compared with a control group of children treated with conventional radiotherapy for cranial and abdominopelvic tumors [39].

The methods that allow the intensity modulation of the radiation beams increase the volume of tissue receiving low dose compared to conventional radiotherapy [40]. The effects in adult patients are the same, however, there are 3 reasons for special consideration in the treatment of children: higher sensitivity to radiation induced cancer, relation of scattered dose to the small body volume and genetic susceptibility due to germline mutations [18,41-45]. While high dose to neighbouring structures can be selectively decreased by the means of IMRT, low dose is distributed in the rest of the body. Consequences of this special treatment technique can only be estimated until now.

Data of the childhood cancer survivor study (CCSS) showed 5 year survival rates of 79% for all different tumor entities [46]. With such a high number of long term survivors secondary neoplasms become highly relevant. The risk is especially increased in patients of very young age, Hodgkin's disease, treatment with alkylating agents, radiation therapy and female gender [47,48].

Secondary cancer induction is dose dependent and tissue irradiated with doses below 6 Gy is known to be especially endangered to develop secondary cancer [49]. The calculated risk of secondary malignancies after treatment with IMRT was estimated to be doubled [17,19]. It is important to note that these numbers are only estimations and calculations with no fundament of clinical data due to the lack of enough follow-up time. In addition integral dose is often discussed to be potentially higher in IMRT compared to conventional radiotherapy. This is not necessarily true since the high dose region to normal tissue is markedly reduced with the improved conformity [50]. As stated above the characteristic new feature of dose exposure in IMRT is a shift towards low dose spread out. Especially in the tissues with a high incidence of secondary cancers the ability of IMRT to produce conformal avoidance of these structures might limit the risk of these late effects. Techniques like helical tomotherapy have the potential of selectively sparing the thyroid gland and breast tissue in craniospinal irradiation.

The number of children treated with IMRT and the hard evidence for the benefit of this technology is limited [13]. However, waiting for this evidence would last for many years. Many of the uncertainties cannot be answered by simply transferring the standards of evidence based medicine in medical oncology one by one to radiation oncology. Randomizing children or adults in two different radiotherapy regimens knowing that one will definitely inactivate the parotid glands, one kidney or affect bone growth is simply unethical. Withholding children the possibility to reduce doses to organs at risk in difficult cases is hard to justify. As long as proton treatment with its great potential of decreased integral dose is not widely available, IMRT provides an excellent tool in difficult situations. Patient selection is absolutely crucial with regard to the worries about potentially increased chances of secondary malignancies. Reserved for complex cases with close proximity of organs at risk IMRT represents a powerful and versatile treatment option when used with the necessary caution [25,51].

## Conclusion

Intensity modulated radiotherapy is a feasible method of radiotherapy for paediatric malignancies. It was applied safely in 31 patients within the last eight years in difficult oncologic situations. Conventional radiotherapy would have been associated with limited dose to the target or high normal tissue complication probability. In all the presented patients it was decided that the benefit of increased tumor control probabilities and improved sparing of organs at risk had a higher clinical impact than the calculated increased risk of late side-effects.

As long as the risk of secondary cancer induction can only be estimated IMRT for children should only be used with caution. Longer follow up time is needed to quantify this long term complication. Conventional radiotherapy remains the standard of care in radiation oncology for children and can be delivered with acceptable toxicity in the majority of children.

Nevertheless, reserved to special cases with close proximity of sensitive structures, it can provide great benefit for paediatric patients and should not be withheld because of estimations based on a radiobiological model. It widens the therapeutic window and reduces long term toxicity for an increased number of long term cancer survivors.

## Declaration of competing interests

The authors declare that they have no competing interests.

## Authors' contributions

FS is responsible for data acquisition, literature research and writing of the manuscript. ES is responsible for data acquisition, statistical analysis and writing of the manuscript. SN is responsible for the physical aspects of IMRT planning and treatment of the children. HB is responsible for the anaesthesia management of the children. PH is responsible for the clinical treatment of the children as head of the division of radiation oncology in the German Cancer Research Center. JD is responsible for the clinical treatment of the children as of the department of radiation oncology in the University of Heidelberg. MM is responsible for the medical aspects of treatment planning and application, idea for this paper, literature research and proof reading. All authors read and approved the final manuscript.
